# Nutrigenomic Analysis of Diet-Gene Interactions on Functional Supplements for Weight Management

**DOI:** 10.2174/138920208784533638

**Published:** 2008-06

**Authors:** Francis C Lau, Manashi Bagchi, Chandan Sen, Sashwati Roy, Debasis Bagchi

**Affiliations:** 1 InterHealth Research Center, Benicia, CA, USA; 2 Laboratory of Molecular Medicine, Department of Surgery, Ohio State University Medical Center, Columbus, OH, USA; 3 Department of Pharmacy Sciences, Creighton University Medical Center, Omaha, NE, USA

**Keywords:** Insulin resistance, glucose tolerance factor, supplemental chromium, Garcinia cambogia, (-)-hydroxycitric acid, overweight, obesity, diabetes, cardiovascular disease, nutritional interventions, microarrays, nutrigenomics.

## Abstract

Recent advances in molecular biology combined with the wealth of information generated by the Human Genome Project have fostered the emergence of nutrigenomics, a new discipline in the field of nutritional research. Nutrigenomics may provide the strategies for the development of safe and effective dietary interventions against the obesity epidemic. According to the World Health Organization, more than 60% of the global disease burden will be attributed to chronic disorders associated with obesity by 2020. Meanwhile in the US, the prevalence of obesity has doubled in adults and tripled in children during the past three decades. In this regard, a number of natural dietary supplements and micronutrients have been studied for their potential in weight management. Among these supplements, (–)-hydroxycitric acid (HCA), a natural extract isolated from the dried fruit rind of Garcinia cambogia, and the micronutrient niacin-bound chromium(III) (NBC) have been shown to be safe and efficacious for weight loss. Utilizing cDNA microarrays, we demonstrated for the first time that HCA-supplementation altered the expression of genes involved in lipolytic and adipogenic pathways in adipocytes from obese women and up-regulated the expression of serotonin receptor gene in the abdominal fat of rats. Similarly, we showed that NBC-supplementation up-regulated the expression of myogenic genes while suppressed the expression of genes that are highly expressed in brown adipose tissue in diabetic obese mice. The potential biological mechanisms underlying the observed beneficial effects of these supplements as elucidated by the state-of-the-art nutrigenomic technologies will be systematically discussed in this review.

## 1 INTRODUCTION

With the success of the Human Genome Project and the advances in molecular biology, a new discipline, namely nutrigenomics, in the field of nutrition research has emerged [1, 2]. This new wave of molecular nutrition, which unfolds the mystery behind nutrition-gene interactions, may provide the strategies for developing safe and effective nutritherapies for individuals such as those who are under siege by the obesity epidemic [3, 4]. The World Health Organization (WHO) coined the term globesity to signify the fact that obesity has rapidly grown into a global epidemic in recent years [5-7]. According to WHO, more than 60% of the global disease burden will be attributed to chronic disorders associated with obesity in the year 2020 [8]. Meanwhile in the US, the prevalence of obesity has doubled in adults and tripled in children during the past three decades [9-12]. Currently, the number of overweight and obese US adults surpasses that of the normal weight US adults for the first time in history [13, 14]. This trend is expected to continue at an alarming rate with an estimated annual increase of up to 0.9% [15]. As a result, it is projected that 75% of the US adults will be overweight or obese by the year 2015 [15].

Since food is the central component contributing to obesity, a long-term lifestyle change in diet in conjunction with exercise may present a cost-effective first-line of intervention for obesity [16, 17]. To reflect the need for individualized nutritional plans, the United States Department of Agriculture (USDA) has recently replaced its Food Guide Pyramid with MyPyramid emphasizing on whole grains, fruits and vegetables [18]. Indeed, there are a number of natural dietary supplements and trace elements that may be used as a part of nutritional lifestyle intervention for weight management [19]. In this regard, (–)-hydroxycitric acid (HCA), a natural extract isolated from the dried fruit rind of Garcinia cambogia, and the trace metal chromium(III) have been shown, in animal models and clinical trials, to be safe and efficacious as weight loss supplements [20, 21]. This chapter will discuss the potential biological mechanisms underlying the observed beneficial effects of these supplements as elucidated by the state-of-the-art nutrigenomic technologies.

## 2 NUTRIGENOMICS: THE NEW FRONTIER OF NUTRITIONAL SCIENCE

### 2.1 Definition and Scope of Nutrigenomics and Nutrigenetics

Nutrigenomics is the study of the effect of nutrition or dietary components on the transcriptome of cells and tissues. The broad scope of nutrigenomics involves studying the effect of nutrition or dietary components on the structure, integrity and function of the genome. It is an offshoot of the science of genomics, and is being shaped by the evolving and powerful genomic technologies. It helps to understand how bioactive dietary components influence the human genome by altering the transcriptome or gene expression profile [22]. 

The term nutrigenetics is sometimes used interchangeably with nutrigenomics. However, nutrigenetics is the retrospective investigation of how genetic variations such as single nucleotide polymorphisms (SNPs) in haplotype maps (HapMap) give rise to differences in response to specific nutrients and eventually lead to differences in health and disease status among individuals [23, 24]. Fig. (**1**) illustrates the distinctions between nutrigenomics and nutrigenetics in simple terms. This chapter will focus on the application of nutrigenomics to weight management.

### 2.2 Nutrigenomics and Obesity

Accumulating evidence has indicated that dietary components not only fuel the body but also participate in the modulation of gene expression [25]. Therefore, nutrigenomics represents the future of nutritional research. In fact, recent surveys have projected that 33% of the US consumers will be collecting and acting on nutrigenomic information by the year 2010 [26].

One of the pressing issues faced by the nutrition research community is the obesity epidemic. Nutrigenomics approaches have been initiated for obesity research and the data from each of these studies will provide new insights into the mechanisms of nutrient-gene interaction [27]. 

#### 2.2.1 Obesity Epidemic and Socioeconomic Burden

The World Health Organization (WHO) defines overweight and obese by body-mass index (BMI, kg/m^2^) cutoff points of 25 and 30, respectively [15]. Globally, it is estimated that 1.6 billion adults are overweight while 400 million are obese [28]. In the US, the estimated number of overweight and obese adults has recently surpassed that of their normal weight counterparts [1, 15, 29]. This alarming increase in the prevalence of obesity may be attributable to lifestyle changes in modern society favoring decreased activity and increased food consumption, thus creating a fertile obesogenic environment [7]. The national financial impact from obesity epidemic is tremendous. According to the Surgeon General, the direct and indirect costs of obesity were approximately $120 billion in the turn of the millennium [30].

#### 2.2.2 Obesity Etiology 

Although the fundamentals of energy imbalance in obesity are well accepted, the complex factors affecting this disequilibrium remain unclear [7, 31]. 

Even though it is quite inconceivable to entertain the notion that obesity might be caused by infectious agents, several studies have indicated that human adipogenic adenoviruses and human gut microbes such as Bacteriodetes and Firmicutes may represent additional contributing factors to human obesity [32-36]. Thus, the etiology and pathogenesis of obesity may comprise a myriad of hitherto unknown factors that influence the imbalance of energy consumption and its expenditure. 

#### 2.2.3 Obesity-Related Disorders 

The consequences of obesity are that they significantly decrease the quality of life and life expectancy while greatly increase the risk for a number of diseases related to increased morbidity and mortality [37-39]. An estimated loss of life expectancy by 7 years has been attributed to obesity at age 40 [38].

The mounting challenge of obesity epidemic is its association with a broad spectrum of metabolic disorders such as type II diabetes and cardiovascular disease, and certain cancers including colon cancer and breast cancer [40, 41]. The statistics for the correlation of obesity to these disorders are staggering: 80% of type II diabetes, 70% of cardiovascular diseases, and 42% of breast and colon cancer have been linked to obesity [21, 42, 43]. Also, 85% of the children diagnosed with type II diabetes are classified as obese [21]. Indeed, the dependency of type II diabetes on obesity is so strong that the term “diabesity” has been coined for this phenomenon [44, 45]. Hence, elevated BMI results in significant increases in the disease risks and health care costs [46, 47].

#### 2.2.4 Weight Management 

Accumulating evidence indicates that obesity-related risk factors are not only preventable but also ameliorable through weight loss and long-term weight management programs [48-50]. Since the root of this ever-growing obesity epidemic seems to link intimately with increased food consumption, the therapeutic goals for weight loss and weight management are to curb appetite, reduce food intake, and decrease fat absorption and increase fat oxidation [17, 51]. Three key strategies of weight interventions are commonly used worldwide to combat the obesity epidemic [46]: (1) bariatric surgery, which is an effective weight loss procedure for people suffering from severe clinical obesity, (2) pharmacotherapy, which is the use of anti-obesity drugs, such as rimonabant, phentermine, sibutramine, and orlistat [52, 53], and (3) life-style interventions, which include diet and exercise, as well as use of various anti-obesity nutritional supplements which have been thoroughly studied and have been proved to be safe and efficacious. 

In the following text, the discussion will be restricted to two such nutritional supplements that have been used for weight management. These are niacin-bound chromium(III) complex (ChromeMate®, NBC) and (–)-hydroxycitric acid (Super CitriMax®, HCA-SX). 

A number of biochemical, pharmacological and toxicological studies have been conducted to show that niacin-bound chromium(III) complex as a novel micronutrient is capable of reducing body fat mass while increasing lean body mass [20, 54]. The (–)-hydroxycitric acid (HCA) is a natural extract from the dried fruit rind of Garcinia cambogia. Numerous studies have demonstrated that a novel calcium-potassium double salt of 60% HCA preparation (HCA-SX) is safe, bioavailable and efficacious in promoting healthy body weight. 

#### 2.2.5 Nutritional Supplements

There is a plethora of weight management dietary supplements on the market. The common claims of these products include increase in energy expenditure, satiety and fat oxidation, and decrease in fat absorption. However, the efficacy and safety for many of these dietary supplements have not been systematically and scientifically investigated. Therefore, the U.S. Food and Drug Administration (FDA) has taken regulatory actions against a number of dietary supplements.

On the contrary, a number of biochemical, pharmacological and toxicological studies have been conducted to show that niacin-bound chromium(III) complex is a novel micronutrient that is capable of reducing body fat mass while increasing lean body mass [20, 54]. NBC has also been shown to be effective in diminishing the obesity-related risk factors of metabolic syndrome by promoting glucose-insulin sensitivity [55-57]. The importance of chromium(III) was revealed five decades ago with the discovery that chromium(III) is the central component of the biologically active form of glucose tolerance factor (GTF) found in Brewer’s yeast [58, 59]. GTF prevented diabetes in experimental animals by potentiating the action of insulin and modulating protein, fat and carbohydrate metabolism [60]. 

A comparative clinical study evaluating the effects of chromium(III) supplements (NBC and chromium(III) picolinate) with or without exercise training in young, obese women showed that exercise training combined with NBC supplementation was associated with significant weight loss and lowered insulin response to an oral glucose load [61]. In contrast, chromium(III) picolinate supplementation resulted in a significant weight gain. Therefore, NBC supplementation combined with exercise training proved to be beneficial for weight loss and for the reduction of risk factors associated with diabetes [61].

A subsequent randomized, double-blind, placebo-controlled, crossover study on 20 overweight African-American women has showed that subjects taking 200 µg NBC three times daily exhibited a significant fat loss without causing a reduction in lean body mass as compared to subjects receiving placebo [62]. 

The other extensively studied weight management herbal supplement is the (–)-hydroxycitric acid (HCA), a natural extract from the dried fruit rind of Garcinia cambogia. Numerous studies have demonstrated that a novel calcium-potassium double salt of 60% HCA preparation (HCA-SX) is safe, bioavailable and efficacious in promoting healthy body weight. The unique structural characteristics of HCA-SX make it completely water soluble and highly bioavailable, as well as tasteless, odorless and colorless in solution [63-70]. 

A randomized, placebo-controlled, double-blind study on the beneficial effect of HCA-SX was conducted in 54 male and female subjects who were 15-45% overweight [71]. The subjects in the treatment group (n=30) of this eight-week study were given Lipodex-2™ (500 mg of Garcinia cambogia extract and 100 µg elemental chromium as NBC) 3 times a day and the subjects in the control group (n=24) received the placebo. All subjects were encouraged to adhere to a low-fat diet plan (1,200 kcal diet/day) and to drink 64 oz of water per day. The participants in the treatment group lost an average of 11.14 pounds/person while those in the control group lost an average of 4.2 pounds/person [71].

A subsequent eight-week randomized, placebo-controlled, double-blind study was conducted in sixty obese subjects [72]. Subjects received either the placebo (n=30) or HCA (n=30). The dose for HCA was 400 mg three times daily 30 minutes before each meal. All subjects were on a low-fat diet of 1,200 kcal/day and were instructed to exercise 3 times a week for 8 weeks. The HCA-supplemented group exhibited a significant weight loss as compared to the placebo group. The composition of the weight loss, determined by the near infra-red (NIR) technique, showed that 87% of the weight loss in the HCA group is due to fat loss. Appetite scores were also significantly reduced in the HCA group [72]. 

The underlying molecular mechanisms for the observed benefits of NBC and HCA-SX on weight loss were largely unclear. However, the advent of nutrigenomics opens up a new avenue for the elucidation of nutrition-gene interactions that will aid in the design of new nutritherapeutic strategies for weight management.

## 3 APPLICATION OF NUTRIGENOMICS IN WEIGHT MANAGEMENT

### 3.1 Chromium(III)-Gene Interaction 

Numerous animal and human clinical studies have established the safety and efficacy of chromium(III) supplementation in combating insulin resistance, reducing body fat and increasing lean body mass [20, 54]. However, the underlying molecular mechanism for the observed beneficial effects of chromium(III) is unclear. In contrast, the structure, function, and mode of action of the majority of other essential trace elements such as copper and zinc have been well characterized. Even though there have been several attempts to identify the specific organic chromium(III) complex that exhibits biological functions, the results have generally been controversial at best [73-76]. 

In order to decipher the mechanism modulating the genetic response to NBC-supplementation, a recent study utilized the high-throughput screening (HTS) technology to examine NBC-induced alteration in gene expression profiles (transcriptome) [77]. This study investigated the effect of oral NBC supplementation on the physiological parameters of obese mice homozygous for Type II diabetes spontaneous mutation (Lepr^db^), and the alteration in transcriptome of subcutaneous adipose tissues in these obese mice [77]. Supplementation regimen was carried for 10 weeks in male Lepr^db^ mice which were randomly divided into the NBC (n=7, NBC) or placebo (n=7, PBO) group. 

Blood samples were drawn from the mice before (baseline) and after 6 weeks of supplementation. Blood glucose level as well as lipid profile parameters such as total cholesterol (TC), HDL cholesterol (HDLC), triglycerides, LDL, and the TC-to-HDLC ratio were assessed at week 6 and compared to baseline data collected before any supplementation. After 8 weeks of supplementation, oral glucose tolerance test (OGTT) was performed by challenging the mice with 1.5 mg/g body weight of glucose solution and measuring blood glucose levels at 30, 60, and 120 min after glucose challenge [77]. At 10 weeks post-supplementation, mice were euthanized and subcutaneous fat was removed for isolation of RNA. The quality of RNA was verified before the synthesis of targets from RNA for hybridization to probes (41,101 probe sets) on the mouse genome microarrays (430 v2.0). 

The results of biochemical tests, as summarized in Table **1**, showed that NBC-supplementation significantly attenuated the levels of triglycerides, TC, LDL cholesterol, and TC-to-HDLC ratio in the plasma of the obese diabetic mice. The plasma level of HDLC in these mice was significantly increased by NBC-supplementation. OGTT findings indicated a significant enhancement by NBC-supplementation in the rate of blood glucose clearance from 60 to 120 min after glucose challenge [77]. The observations from this study agreed with the previous findings in other human and animal studies that NBC supplementation play a beneficial role in glucose and lipid metabolism [77]. 

NBC-induced changes in the transcriptome of subcutaneous adipose tissues of these obese diabetic mice were interrogated by an unbiased genome-wide microarray approach in an attempt to identify candidate genes whose expressions were sensitive to NBC-supplementation [77]. The data-mining scheme is presented in Fig. (**2**) where, among the 45101 probe-sets interrogated, only a small subset of genes was found to be influenced by NBC-supplementation. The overall effect of NBC-supplementation on the genome of the adipose tissues was positive since it stimulated more genes than it inhibited them as illustrated by Fig. (**3**). The NBC-induced genes are known to be involved in glycolysis, muscle metabolism, and muscle development. The expression of muscle-specific genes in fat tissue over time has been shown to reduce fat content in the adipose tissues [78]. The NBC-suppressed genes in the adipose tissues are known to play important roles in thermogenic process of brown fat tissue. On the whole, the microarray data indicated that NBC-supplementation did not induce a genome-wide perturbation; rather, NBC-supplementation specifically influenced a small subset of genes that are biologically relevant to adipocyte maintenance. 

As shown in Fig. (**3**), bioinformatic analyses of the microarray data resulted in 161 up-regulated and 91 down-regulated genes by NBC-supplementation in obese diabetic mice. The expression of several biologically relevant candidate genes was further verified by real-time PCR and the results are summarized in Table **2**. Enolase 3 (ENO3) showed the highest up-regulation (7.6-fold) compared to controls in the fat tissues, in response to NBC-supplementation. Enolase is a dimeric glycolytic enzyme that catalyzes the interconversion of 2-phosphoglycerate and phosphoenolpyruvate. Beta-enolase subunit is encoded by ENO3 gene and is responsible for more than 90% of the enolase activity in adult human muscle [79]. It has been shown that mutations in ENO3 led to β-enolase deficiency that resulted in defects in glycolysis and metabolic myopathies [79]. Another glycolytic gene, encoding the enzyme glucose phosphate isomerase (GPI), was also up-regulated in the NBC-supplemented obese diabetic mice. It has been documented that glycolytic genes such as ENO3 and GPI are down-regulated in the visceral adipose tissues of morbidly obese patients as compared to non-obese individuals [80]. Calsequestrin, the most abundant calcium-binding protein in the sarcoplasmic reticulum of skeletal and cardiac muscle, was up-regulated by NBC-supplementation. Since chromium has been demonstrated to enhance the expression of plasmalemmal calcium-ATPase in smooth muscle cells, it is possible that chromium may influence calcium homeostasis by increasing the calcium storage capacity through up-regulation of calsequestrin [81, 82]. Tropomyosin-1 (TPM1) gene, which encodes for the α-subunit (α-tropomyosin) of the tropomyosin family of proteins, was up-regulated in response to NBC-supplementation. TPM1 protein plays an important role in calcium-dependent regulation of striated muscle contraction [83-85]. Expression of these up-regulated genes in fat tissue over time has been shown to attenuate the fat content of the tissue [78]. 

The NBC-induced down-regulated genes included adipocyte-specific genes such as the cell death-induced DNA fragmentation factor (CIDEA), mitochondrial uncoupling protein 1 (UCP1) and tocopherol transfer protein (TTP) (Table **2**). TTP protein is involved in the transport of α-tocopherol from hepatocytes into peripheral tissues including adipose tissue [86]. Since α-tocopherol severs as a potent antioxidant, down-regulation of TTP may decrease the lipid-phase antioxidant defense in the adipose tissue thus promoting the breakdown of adipose tissues [77]. In addition, because α-tocopherol readily interconverts into lipoproteins and TTP is likely to facilitate the incorporation of α-tocopherol into LDL [87, 88], down-regulation of TTP in the adipose tissues is expected to lower the levels of LDL. Interestingly, the physiological findings indicated that the plasma levels of LDL were indeed reduced as indicated in Table **1**. Both CIDEA and UCP1 proteins are highly expressed in brown adipose tissue (BAT) and they play important roles in the thermogenesis and energy expenditure of BAT [89, 90]. CIDEA-knockout mice exhibited resistance to diet-induced obesity and diabetes [90]. Thus, down-regulation of CIDEA by NBC may exhibit similar effects, that is, weight loss and resistance to diet-induced obesity. 

Taken together, the data suggest that NBC exerts its beneficial effects of weight loss through regulation of specific genes in the fat cells of obese diabetic mice [90]. Such genomic approach to nutritional research has paved the way for future investigations to unravel the molecular basis of chromium-gene interactions. 

### 3.2 HCA-Gene Modulation

#### 3.2.1 Animal Study 

The effects of low-dose oral HCA-SX on the body weight and abdominal fat gene expression profile of Sprague Dawley rats were investigated [91]. The rats were randomly divided into the HCA-SX group and the control group; animals in the HCA-SX group were supplemented with 10 mg HCA-SX/kg body weight for eight weeks after which they were sacrificed. Plasma samples were collected for the assessment of leptin level by ELISA, and abdominal fat was removed for microarray studies. No overt behavioral changes were observed in rats from the HCA-SX group compared to controls; however, a significant weight loss was observed in the HCA-SX group starting from week six and continued until the end of the HCA-SX supplementation period (week eight). Plasma leptin levels were not affected by HCA-SX supplementation, but transcription of the leptin gene in the abdominal fat cells from the HCA-SX group, as indicated by real-time RT-PCR, was significantly attenuated [91].

Genome-wide changes in gene expression profile following HCA-SX treatment was studied using DNA microarray. Rat genome arrays containing 15,923 probe sets were screened for alterations in the transcriptome of abdominal fat induced by HCA-SX feeding. dChip-assisted [92] analysis of the expression data resulted in 93 up-regulated and 18 down-regulated genes [91]. Three relevant HCA-SX–induced up-regulated genes selected for further validation by real-time RT-PCR analysis were prostaglandin D synthase (PDS), aldolase B (AldB), and lipocalin (LCN2). The data from real-time RT-PCR were in agreement with DNA microarrays [91]. To put these HCA-SX up-regulated genes into perspective as to how they could be modulated in specific signaling pathways, they were mapped to known pathways related to fat metabolism. Several bioinformatics tools such as GenMapp, KEGG (Kyoto Encyclopedia of Genes and Genomes) and Gene Ontology were used to reconstruct the pathways. This approach overlapped the candidate genes with the monoamine G-protein-coupled receptor pathway. Specifically, it was found that the serotonin receptor expression was consistently up-regulated in response to HCA-SX supplementation in the abdominal fat. This discovery is consistent with the previous observation of the serotonergic property of HCA-SX that HCA-SX promotes serotonin release form isolated rat brain [93].

One important finding was that HCA-SX supplementation selectively induced a small subset (approximately 1%) of all the genes screened in the abdominal fat. The expression of vital genes required for fundamental support of cellular functions was not affected by HCA-SX supplementation. Furthermore, the changes in the expression profile did not indicate any discernible stress or toxicity response to HCA-SX supplementation [91]. Therefore, the results thus far concur with prior findings to support the effective and safe use of HCA-SX in weight loss and validated the previous observation for the serotonergic property of dietary HCA-SX supplementation [93].

#### 3.2.2 Human Adipocyte Study

Defects in the lipolytic process have been linked to human obesity [94]. Roy *et al*. (2007) sought to investigate the effects of HCA-SX on lipolysis in cultured human adipocytes using biochemical and genomic approaches [95]. Subcutaneous preadipocytes isolated from obese women were allowed to differentiate to adipocytes for two weeks in culture. The effects of HCA-SX on lipid metabolism and transcriptomic profile were examined. HCA-SX treatment at the experimental conditions did not cause any cytotoxicity in the human adipocytes. Fig. (**4**) showed that HCA-SX treated adipocytic cells exhibited a significant increase in the fat droplet dispersion as compared to the control cells suggesting that HCA-SX treatment potentiated lipolysis through mobilization of triacylglycerol storage.

In order to gain insight into the genome-wide effects of HCA-SX treatment on human adipocyte gene expression profile, human genome microarrays were used to compare the changes in the transcriptomes between the HCA-SX treated and control human adipocytes. The data acquisition and analysis processed were illustrated in Fig. (**5**). The human genome microarrays containing a total of 54,676 probe sets per chip were use to screen HCA-SX induced alterations in gene expression profiles of the human adipocytes. HCA-SX treatment caused statistically significant changes in the transcriptome of a very small cluster of genes of which 348 genes were significantly down-regulated while 366 genes were significantly up-regulated as demonstrated in Fig. (**6**). 

The expression of selected HCA-SX responsive genes obtained by microarray analysis was independently verified by real-time RT-PCR (Table **3**). Of these, the down-regulated perilipin gene was of particular interest. Perilipin protein, the product of the perilipin gene, coats the surfaces of lipid droplets in adipocytes and is found at lower levels surrounding lipid droplets in steroidogenic cells. Perilipin proteins drive triacylglycerol storage in adipocytes by regulating the rate of basal lipolysis [96]. HCA-SX–induced suppression of perilipin gene expression is likely to contribute to the observed lipolytic effect of HCA-SX on lipid droplet dispersion as illustrated in Fig. (**4**).

Peroxisome proliferator-activated receptor (PPAR) gamma co-activator 1-alpha (PGC1-alpha) was also found to be down-regulated by HCA-SX in human adipocytes. PGC1-alpha is a transcriptional co-activator protein that coordinately regulates metabolic pathways; thus, it may play an important role in the pathogenic conditions such as obesity, diabetes, and cardiomyopathy [97]. It has been shown that PGC1-alpha is induced during mitochondrial biogenesis accompanied by adipogenesis to complement the requirement of ATP and acetyl-CoA for lipogenesis [98]. Peroxisomal trans-2-enoyl-CoA reductase (PECR) catalyzes the conversion of phytol to phytanoyl-CoA in peroxisomes. It has been shown that phytanic acid, a derivative of phytol, induces adipocyte differentiation in human pre-adipocytes suggesting that PECR may serve as an activator of retinoid X receptors (RXR) or PPAR [99, 100]. Thus, down-regulation of PGC1-alpha and PECR by HCA-SX treatment may act in concert to promote the anti-adipogenic effect of HCA-SX resulting in weight loss.

HCA-SX treatment also significantly decreased the expression of endothelial lipase (LIPG) gene in human adipocytes. Over-expression of LIPG decreases the plasma level of high-density lipoprotein (HDL) cholesterol. Since HDL level is inversely associated with risk of atherosclerotic cardiovascular disease, reduction in HDL plasma level is likely to produce adverse effects that augment the risk factors for cardiovascular diseases [101]. In addition, HDL hydrolysis by endothelial lipase (gene product of LIPG) activates PPARα which in turn induces inflammation [102]. Therefore, down-regulation of LIPG by HCA-SX may be beneficial for lipoprotein metabolism and vascular health.

Elongation of very-long-chain fatty acids-like 3 (ELOVL 3) gene was suppressed in human adipocytes by HCA-SX treatment. ELOVL3 protein is involved in fatty acid biosynthesis [103]. It has been shown that ELOVL3 gene expression is significantly induced during adipogenesis. The gene product of ELOVL3 catalyzes the synthesis of very-long-chain fatty acids and triglycerides in the adipose tissues, thus facilitating the process of adipogenesis [104]. Therefore, suppression of ELOVL3 in the human adipocytes by HCA-SX treatment may inhibit adipogenesis in fat tissues [95]. 

HCA-SX significantly reduced the expression of cytoplasmic epoxide hydrolase 2 (EPHX2) gene. EPHX2 gene product is involved in the metabolism of arachidonic acid [105]. Polymorphisms in EPHX2 gene have been linked to the pathogenesis of coronary heart disease (CHD) and atherosclerosis; therefore, EPHX2 has been implicated as a potential cardiovascular disease-susceptibility gene [106, 107]. Since obesity is a major risk factor for CHD and atherosclerosis, down-regulation of EPHX2 by HCA-SX in human adipocytes may exert protective effect against these cardiovascular diseases.

Several members of the matrix metalloproteinase (MMP) family of protease genes were up-regulated in human adipocytes by HCA-SX treatment (Table **3**). Specifically, the expression of genes encoding MMP1, 3, and 10 proteases was stimulated by HCA-SX treatment. MMP proteases play important role in matrix protein turn-over and tissue remodeling. MMP1 (or interstitial collagenase) and MMP10 (or stromelysin 2) play important role in fibrillar collagen and proteoglycan metabolism [108]. Proteoglycan turnover has been shown to be involved in adipocyte degradation and weight loss [109, 110]. MMP3 (or stromelysin 1) has been shown to activate MMP1 and impair adipose tissue development [111, 112]. Low levels of stromelysins (such as MMP3 and MMP10) have been linked to obesity [111, 113]. MMP10 degrades extracellular matrix thereby negatively regulates angiogenesis and vascular remodeling [114]. Since angiogenesis is functionally linked to adipocyte development, the anti-angiogenic property of MMP10 may indirectly inhibit adipogenesis in the human adipocytes [115]. Induction of these MMP proteases by HCA-SX treatment may exert an anti-angiogenic and anti-adipogenic effect on the human adipocytes leading to the observed fat droplet dispersion as illustrated in Fig. (**4**).

Tissue-type plasminogen activator (PLAT) regulates fibrinolysis and is the primary cellular defense mechanism against thrombosis [116]. Elevated risk of arterial thrombosis is associated with adiposity. It has been shown that the release of PLAT from the endothelium of obese and overweight adults is significantly reduced [116]. The finding from the current study showed that treatment of human adipocytes with HCA-SX increased the expression of PLAT gene suggesting that HCA-SX may contribute to the restoration of endothelial fibrinolytic dysfunction associated with overweight and obesity.

The adipocyte-derived hormone leptin together with its receptor are the major components in the maintenance of energy balance [117, 118]. Disruption of leptin signaling pathway through the deletion of leptin receptor has resulted in obesity [119]. A large body of evidence also indicates that leptin plays important functions in fat oxidation, weight loss, and cardioprotection [120, 121]. Microarray analysis, confirmed by real-time RT-PCR, showed that the expression of leptin in the human adipocytes was significantly up-regulated by HCA-SX treatment.

Taken together, the animal as well as the human adipocyte studies utilizing the nutrigenomic approach demonstrated for the first time the molecular link between the observed anti-lipolytic and anti-adipogenic effects of HCA-SX supplementation and the genomic response to such supplementation. 

## 4 CONCLUSION

Until recently, nutritional research has suffered from the incapability of delineating the mechanisms of diet-gene regulations. The advent of nutrigenomics has provided the tools for the understanding of diet-gene interaction at the molecular level. Accumulating physiological evidence has indicated safe and efficacious use of NBC and HCA-SX in weight management. However, it is not until recently that gene-modulation by NBC and HCA-SX, as revealed by nutrigenomics approach, has begun to come to light. These physionutrigenomics studies represent a novel and powerful approach in nutritional sciences which not only elucidates the underlying molecular mechanisms but also evaluates the health and safety concerns related to the consumption of these dietary supplements. Indeed, cDNA microarray technology provides an extremely efficient means to assess the genome-wide alterations in transcriptomes of biological systems in response to any given therapeutic regimen [122]. In this regard, physionutrigenomics holds the promise to facilitate the design and development of novel and safe nutraceuticals and functional foods to combat obesity and other metabolic disorders.

In addition, nutrigenomics is beginning to pave the way for personalized nutrition as more associations between dietary components and gene regulations are being identified by this functional genomics approach. Moreover, through further understanding of the biological impacts of nutrient-gene modulations, nutrigenomics will provide key insights into the pathogenesis and progression of diet-related disorders.

Nutritherapeutics combined with a healthy life-style may be the most cost-effective and organic way to combat obesity epidemic. Through systematic physiological and biochemical approaches, the safety and efficacy of NBC and HCA-SX in weight management have been demonstrated in chapter. The studies discussed here also offer unequivocal evidence that it is possible to use the powerful tool of nutrigenomics to decipher the molecular basis for the observed beneficial effects of dietary supplements.

## Figures and Tables

**Fig. (1):**
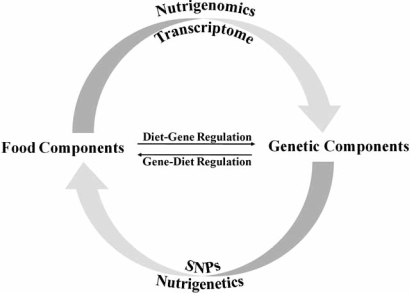
Distinctions between nutrigenomics and nutrigenetics. The investigation of how food components modulate changes in gene expression profile or transcriptome is defined as nutrigenomics. One the other hand, nutrigenetics is defined as the study of how genetic variations such as single nucleotide polymorphism (SNP) among individuals affect their response to specific food components.

**Fig. (2):**
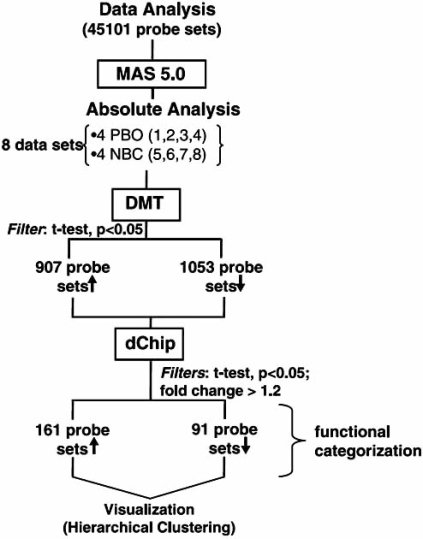
GeneChip^®^ data analysis scheme. Type 2 Lepr^db^ obese diabetic mice were supplemented with NBC (n=4) or with placebo (n=4) for 10 weeks. After 10-week supplementation, mice were euthanized and subcutaneous fat was removed for RNA extraction and microarray analysis. NBC-sensitive genes were identified by GeneChip^®^ mouse genome 430 v2.0 arrays from Affymatrix. Raw data were collected and analyzed with the Affymatrix Microarray Suite 5.0 (MAS) and Data Mining Tool 2.0 (DMT) softwares. The Student’s t-test was performed with DMT on data generated by MAS to identified genes whose expression was significantly changed (P<0.05) in the NBC-supplement group as compared to the control group. DNA-Chip analyzer (dChip) was used for probe-level analysis and creating hierarchical clustering of the post-DMT data with fold changes set at >1.2 and P-value of t-test set at <0.05 [92]. The up (↑) and down (↓) arrows indicate up- and down-regulation of NBC, respectively. Image reproduced with permission from American Physiological Society [77].

**Fig. (3):**
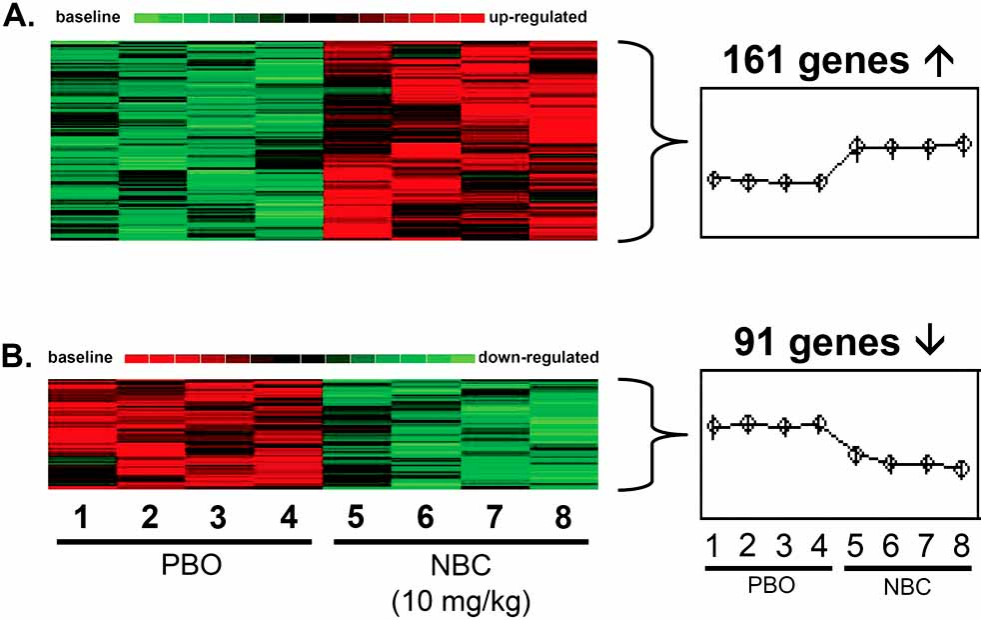
Visualization of hierarchical clustering of genes in response to NBC treatment. Gene expression data were collected as described in Fig. (**4**) legend. Genes whose expression was significantly (P<0.05) altered between the control and the NBC-treated groups were chosen for hierarchical clustering analysis. Expression signal was represented by red to green gradient signifying high to low expression. 161 significantly up-regulated genes (**A**) and 91 significantly down-regulated genes (**B**) were detected. Image used with permission from American Physiological Society [77].

**Fig. (4):**
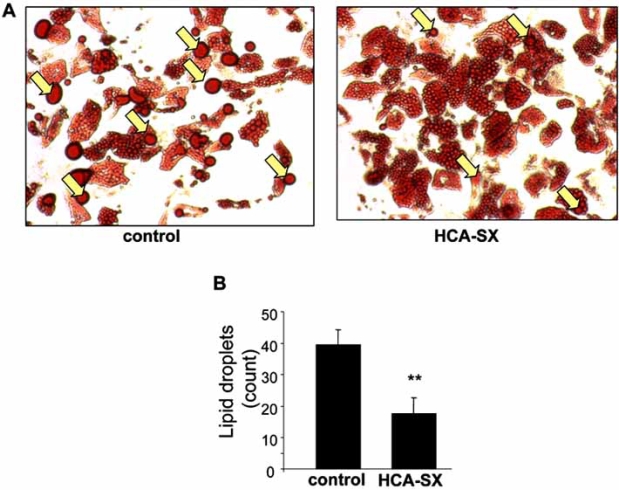
Effect of HCA-SX treatment on the formation of lipid droplets. Differentiated adipocytes from overweight non-diabetic women were cultured with or without HCA-SX (0.5 mg/mL) for 96 hours. After treatment, cells were fixed in 10% buffered formalin, stained with oil red O and images were recorded (**A**). Large oil droplets (indicated by yellow arrows) in each image were counted with Zeiss Axiovision software (**B**). Image reproduced with permission from Mary Ann Liebert, Incorporated [95].

**Fig. (5):**
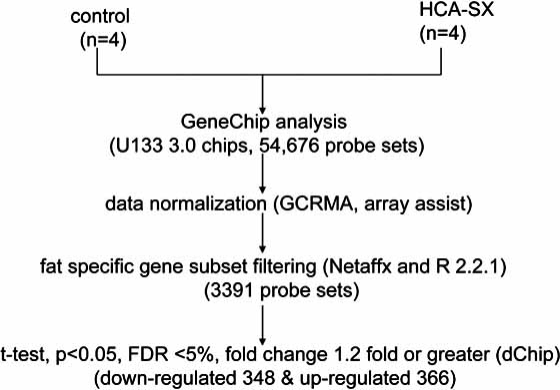
Flowchart of GeneChip^®^ data analysis scheme. RNA was extracted from each sample and used to assess HCA-SX induced alterations in gene expression in human adipocytes with human genome microarray (U133 v2.0). Raw data were collected by GeneChip^®^ operating software. Data contained in the .cel files were normalized by ArrayAssist® Expression software. The NetAffx ™ software was used to identify fat-specific probes. This subset was derived from the main subset with R 2.2.1 software. Genes whose expression was significantly (P<0.05) altered between the two groups were identified by the dChip software with the testing parameter values set as indicated in the diagram. FDR = false discovery rate. Image used with permission from Mary Ann Liebert, Incorporated [95].

**Fig. (6):**
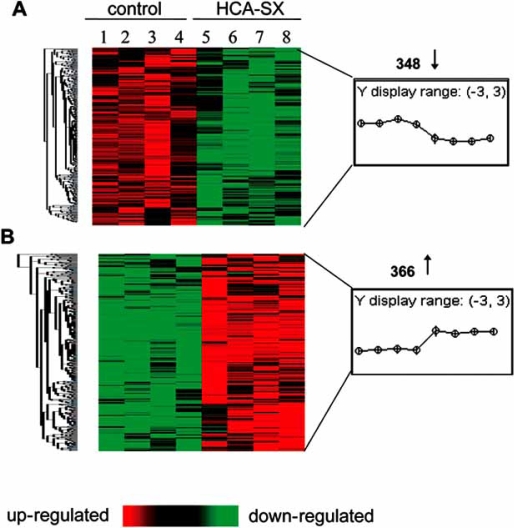
Hierarchical clustering of HCA-SX sensitive genes. Primary human adipocytes were treated with 0.5 mg/mL of HCA-SX (groups 1-4) or untreated (groups 5-8). RNA was extracted from each sample and used to assess HCA-SX induced alterations in gene expression with human genome microarray (U133 v2.0). HCA-SX-responsive genes were identified as described in Fig. (**5**). The genes whose expression was significantly (P<0.05) changed between the control and the HCA-SX-treated groups were used for hierarchical clustering display with the dChip software. Panel **A** represented up-regulated (↑) while panel **B** indicated down-regulated (↓) genes by HCA-SX supplementation. Image used with permission from Mary Ann Liebert, Incorporated [95].

**Table 1:** Comparisons of Blood Lipid Profiles between Placebo Control and Niacin-Bound Chromium (NBC)-Treated Obese Type 2 Diabetic Rats

Parameters (mg/dl)	Placebo	NBC
	(Mean ± SD)	(Mean ± SD)
Total Cholesterol	179 ± 11	144 ± 12**
LDL Cholesterol	83 ± 11	38 ± 12**
Triglycerides	100 ± 21	56 ± 12**
HDL Cholesterol	76 ± 8	95 ± 3**
Total/HDL Cholesterol	2.83 ± 0.31	1.51 ± 0.13**

Lipid profiles were analyzed at week 6 post-supplementation. Data indicate mean ± S.D. with N=7 per group.

** P < 0.0005, indicates statistical significance as compared to the placebo group [77].

**Table 2:** Candidate Genes in Response to NBC-Supplementation

Down-Regulated Genes*	Function
Cell death-inducing DNA fragmentation factor	Lipid metabolism
Uncoupling protein 1	Brown fat thermogenesis
Tocopherol transfer protein	α-tocopherol trafficking
Up-Regulated Genes*	**Function**
Enolase 3	Glycolysis and gluconeogenesis
Calsequestrin 1	Calcium storage, muscle contraction
Tropomyosin 1	calcium-regulated striatal muscle contraction
Glucose phosphate isomerase 1	Glycolysis

Candidate genes were identified by genome-wide microarray analysis. Genes shown in the table represent those whose altered expression was confirmed by quantitative real-time RT-PCR

* (P<0.05, as compared to control) [77].

**Table 3:** HCA-SX Specific Candidate Genes in Human Adipocytes

Down-Regulated Genes*	Function
Matrix metallopeptidase 1	Matrix protein turnover
Matrix metallopeptidase 3	Matrix protein turnover
Matrix metallopeptidase 10	Matrix protein turnover
Plasminogen activator	Cell migration, tissue remodeling, fibrinolysis
Leptin	Energy balance
Up-Regulated Genes*	**Function**
Perilipin	Regulator of lipid storage
Peroxisome proliferative activated receptor γ co-factor 1α	Adipogenesis
Endothelial lipase	Epoxyeicosatrienoic acid metabolism
Elongation of very-long-chain fatty acids-like 3	Fatty acid biosynthesis
Epoxide hydrolase 2	Arachidonic acid metabolism
Peroxisomal trans-2-enoyl-CoA reductase	Fatty acid biosynthesis

The HCA-SX responsive genes revealed by genome-wide microarray analysis were confirmed by real-time RT-PCR

* (P<0.05, as compared to control) [95].

## References

[1] Ordovas JM (2004). The quest for cardiovascular health in the genomic era: nutrigenetics and plasma lipoproteins. Proc. Nutr. Soc.

[2] Kaput J, Noble J, Hatipoglu B, Kohrs K, Dawson K, Bartholomew A (2007). Application of nutrigenomic concepts to Type 2 diabetes mellitus. Nutr. Metab. Cardiovasc. Dis.

[3] Afman L, Muller M (2006). Nutrigenomics: from molecular nutrition to prevention of disease. J. Am. Diet Assoc.

[4] Ferguson LR (2006). Nutrigenomics: integrating genomic approaches into nutrition research. Mol. Diagn. Ther.

[5] http://whqlibdoc.who.int/trs/WHO_TRS_894_(part1).pdf (Accessed 2000).

[6] http://www.who.int/nutrition/topics/obesity/en/index.html (Accessed 2007).

[7] Caballero B (2007). The Global Epidemic of Obesity: An Overview. Epidemiol. Rev.

[8] Chopra M, Galbraith S, Darnton-Hill I (2002). A global response to a global problem: the epidemic of overnutrition. Bull. World Health Organ.

[9] Ogden CL, Flegal KM, Carroll MD, Johnson CL (2002). Prevalence and trends in overweight among US children and adolescents, 1999-2000. JAMA.

[10] Flegal KM, Carroll MD, Ogden CL, Johnson CL (2002). Prevalence and trends in obesity among US adults, 1999-2000. JAMA.

[11] Hedley AA, Ogden CL, Johnson CL, Carroll MD, Curtin LR, Flegal KM (2004). Prevalence of overweight and obesity among US children, adolescents, and adults, 1999-2002. JAMA.

[12] Bachman KH (2007). Obesity, weight management, and health care costs: a primer. Dis. Manag.

[13] NIH (2000). Clinical Guidelines on the Identification, Evaluation, and Treatment of Overweight and Obesity in Adults: The Evidence Report. Place Published.

[14] http://www.cdc.gov/nchs/fastats/overwt.htm (Accessed July 2007).

[15] Wang Y, Beydoun MA (2007). The Obesity Epidemic in the United States--Gender, Age, Socioeconomic, Racial/Ethnic, and Geographic Characteristics: A Systematic Review and Meta-Regression Analysis. Epidemiol. Rev.

[16] Ross R, Dagnone D, Jones PJ, Smith H, Paddags A, Hudson R, Janssen I (2000). Reduction in obesity and related comorbid conditions after diet-induced weight loss or exercise-induced weight loss in men. A randomized, controlled trial. Ann. Intern. Med.

[17] Blackburn GL (2001). Treatment approaches: food first for weight management and health. Obes. Res.

[18] http://www.mypyramid.gov (Accessed 2007).

[19] Pittler MH, Ernst E (2004). Dietary supplements for body-weight reduction: a systematic review. Am. J. Clin. Nutr.

[20] Bagchi M, Preuss HG, Zafra-Stone S, Bagchi D, Bagchi D, Preuss HG (2007). Obesity: Epidemiology, Pathophysiology, and Prevention.

[21] Zafra-Stone S, Bagchi M, Preuss HG, Grover GJ, Bagchi D, Bagchi D, Preuss HG (2007). Obesity: Epidemiology, Pathophysiology, and Prevention.

[22] Mariman EC (2006). Nutrigenomics and nutrigenetics: the 'omics' revolution in nutritional science. Biotechnol. Appl. Biochem.

[23] Mutch DM, Wahli W, Williamson G (2005). Nutrigenomics and nutrigenetics: the emerging faces of nutrition. FASEB J.

[24] Kaput J, Rodriguez RL (2004). Nutritional genomics: the next frontier in the postgenomic era. Physiol. Genomics.

[25] Corthesy-Theulaz I, den Dunnen JT, Ferre P, Geurts JM, Muller M, van Belzen N, van Ommen B (2005). Nutrigenomics: the impact of biomics technology on nutrition research. Ann. Nutr. Metab.

[26] Gillies PJ (2003). Nutrigenomics: the Rubicon of molecular nutrition. J. Am. Diet. Assoc.

[27] Elliott RM, Johnson IT (2007). Nutrigenomic approaches for obesity research. Obes. Rev.

[28] Ahima RS (2007). Obesity: much silence makes a mighty noise. Gastroenterology.

[29] Gibbs WW (2005). Obesity: an overblown epidemic?. Sci. Am.

[30] http://www.surgeongeneral.gov/topics/obesity/calltoaction/CalltoAction.pdf (Accessed August, 2007).

[31] Haffner S, Taegtmeyer H (2003). Epidemic obesity and the metabolic syndrome. Circulation.

[32] Dhurandhar NV, Kulkarni PR, Ajinkya SM, Sherikar AA, Atkinson RL (1997). Association of adenovirus infection with human obesity. Obes. Res.

[33] Atkinson RL, Dhurandhar NV, Allison DB, Bowen RL, Israel BA, Albu JB, Augustus AS (2005). Human adenovirus-36 is associated with increased body weight and paradoxical reduction of serum lipids. Int. J. Obes. (Lond.).

[34] Whigham LD, Israel BA, Atkinson RL (2006). Adipogenic potential of multiple human adenoviruses *in vivo* and *in vitro* in animals. Am. J. Physiol. Regul. Integr. Comp. Physiol.

[35] Ley RE, Turnbaugh PJ, Klein S, Gordon JI (2006). Microbial ecology: human gut microbes associated with obesity. Nature.

[36] Turnbaugh PJ, Ley RE, Mahowald MA, Magrini V, Mardis ER, Gordon JI (2006). An obesity-associated gut microbiome with increased capacity for energy harvest. Nature.

[37] Livingston EH, Fink AS (2003). Quality of life: cost and future of bariatric surgery. Arch. Surg.

[38] Peeters A, Barendregt JJ, Willekens F, Mackenbach JP, Al Mamun A, d Bonneux L (2003). Obesity in adulthood and its consequences for life expectancy: a life-table analysis. Ann. Intern. Med.

[39] Haslam D (2007). Obesity: a medical history. Obes. Rev.

[40] Shaw DI, Hall WL, Williams CM (2005). Metabolic syndrome: what is it and what are the implications?. Proc. Nutr. Soc.

[41] Haslam DW, James WP (2005). Obesity. Lancet.

[42] Joyal SV (2004). A perspective on the current strategies for the treatment of obesity. Curr. Drug Targets CNS Neurol. Disord.

[43] Walker CG, Zariwala MG, Holness MJ, Sugden MC (2007). Diet, obesity and diabetes: a current update. Clin. Sci. (Lond.).

[44] (1980). From the NIH: Successful diet and exercise therapy is conducted in Vermont for "diabesity". JAMA.

[45] Astrup A, Finer N (2000). Redefining type 2 diabetes: 'diabesity' or 'obesity dependent diabetes mellitus'?. Obes. Rev.

[46] McTigue KM, Harris R, Hemphill B, Lux L, Sutton S, Bunton AJ, Lohr KN (2003). Screening and interventions for obesity in adults: summary of the evidence for the U.S. Preventive Services Task Force. Ann. Intern. Med.

[47] Bray GA (2004). Medical consequences of obesity. J. Clin. Endocrinol. Metab.

[48] Anderson JW, Konz EC (2001). Obesity and disease management: effects of weight loss on comorbid conditions. Obes. Res.

[49] Bray GA (2007). The missing link - lose weight, live longer. N. Engl. J. Med.

[50] Wadden TA, Butryn ML, Wilson C (2007). Lifestyle modification for the management of obesity. Gastroenterology.

[51] Pi-Sunyer FX (2002). The obesity epidemic: pathophysiology and consequences of obesity. Obes. Res.

[52] Schnee DM, Zaiken K, McCloskey WW (2006). An update on the pharmacological treatment of obesity. Curr. Med. Res. Opin.

[53] Bray GA, Ryan DH (2007). Drug treatment of the overweight patient. Gastroenterology.

[54] Zafra-Stone S, Bagchi M, Preuss HG, Bagchi D, Vincent JB (2007). The nutritional biochemistry of chromium(III).

[55] Lefavi RG, Anderson RA, Keith RE, Wilson GD, McMillan JL, Stone MH (1992). Efficacy of chromium supplementation in athletes: emphasis on anabolism. Int. J. Sport Nutr.

[56] Mertz W (1998). Chromium research from a distance: from 1959 to 1980. J. Am. Coll. Nutr.

[57] Shapcott D, Hubert J (1979). Proceedings of the Symposium on Chromium in Nutrition and Metabolism, held in Sherbrooke, Canada, July 13-15, 1979.

[58] Mertz W (1975). Effects and metabolism of glucose tolerance factor. Nutr. Rev.

[59] Mertz W (1993). Chromium in human nutrition: a review. J. Nutr.

[60] Mertz W, Toepfer EW, Roginski EE, Polansky MM (1974). Present knowledge of the role of chromium. Fed. Proc.

[61] Grant KE, Chandler RM, Castle AL, Ivy JL (1997). Chromium and exercise training: effect on obese women. Med. Sci. Sports Exerc.

[62] Crawford V, Scheckenbach R, Preuss HG (1999). Effects of niacin-bound chromium supplementation on body composition in overweight African-American women. Diabetes Obes. Metab.

[63] Preuss HG, Garis RI, Bramble JD, Bagchi D, Bagchi M, Rao CV, Satyanarayana S (2005). Efficacy of a novel calcium/potassium salt of (-)-hydroxycitric acid in weight control. Int. J. Clin. Pharmacol. Res.

[64] Downs BW, Bagchi M, Subbaraju GV, Shara MA, Preuss HG, Bagchi D (2005). Bioefficacy of a novel calcium-potassium salt of (-)-hydroxycitric acid. Mutat. Res.

[65] Shara M, Ohia SE, Schmidt RE, Yasmin T, Zardetto-Smith A, Kincaid A, Bagchi M, Chatterjee A, Bagchi D, Stohs SJ (2004). Physico-chemical properties of a novel (-)-hydroxycitric acid extract and its effect on body weight, selected organ weights, hepatic lipid peroxidation and DNA fragmentation, hematology and clinical chemistry, and histopathological changes over a period of 90 days. Mol. Cell Biochem.

[66] Preuss HG, Bagchi D, Bagchi M, Rao CV, Dey DK, Satyanarayana S (2004). Effects of a natural extract of (-)-hydroxycitric acid (HCA-SX) and a combination of HCA-SX plus niacin-bound chromium and Gymnema sylvestre extract on weight loss. Diabetes Obes. Metab.

[67] Shara M, Ohia SE, Yasmin T, Zardetto-Smith A, Kincaid A, Bagchi M, Chatterjee A, Bagchi D, Stohs SJ (2003). Dose- and time-dependent effects of a novel (-)-hydroxycitric acid extract on body weight, hepatic and testicular lipid peroxidation, DNA fragmentation and histopathological data over a period of 90 days. Mol. Cell Biochem.

[68] Ohia SE, Opere CA, LeDay AM, Bagchi M, Bagchi D, Stohs SJ (2002). Safety and mechanism of appetite suppression by a novel hydroxycitric acid extract (HCA-SX). Mol. Cell Biochem.

[69] Soni MG, Burdock GA, Preuss HG, Stohs SJ, Ohia SE, Bagchi D (2004). Safety assessment of (-)-hydroxycitric acid and Super CitriMax, a novel calcium/potassium salt. Food Chem. Toxicol.

[70] Asghar M, Monjok E, Kouamou G, Ohia SE, Bagchi D, Lokhandwala MF (2007). Super CitriMax (HCA-SX) attenuates increases in oxidative stress, inflammation, insulin resistance, and body weight in developing obese Zucker rats. Mol. Cell Biochem.

[71] Conte AA (1993). A non-prescription alternative in weight reduction therapy. Am. J. Bariatr. Med.

[72] Thom E (1996). Hydroxycitrate (HCA) in the treatment of obesity. Int. J. Obes. Relat. Metab. Disord.

[73] Mertz W (1969). Chromium occurrence and function in biological systems. Physiol. Rev.

[74] Haylock SJ, Buckley PD, Blackwell LF (1983). The relationship of chromium to the glucose tolerance factor. II. J. Inorg. Biochem.

[75] Mirsky N, Weiss A, Dori Z (1980). Chromium in biological systems, I. Some observations on glucose tolerance factor in yeast. J. Inorg. Biochem.

[76] Yamamoto A, Wada O, Ono T (1981). A low-molecular-weight, chromium-binding substance in mammals. Toxicol. Appl. Pharmacol.

[77] Rink C, Roy S, Khanna S, Rink T, Bagchi D, Sen CK (2006). Transcriptome of the subcutaneous adipose tissue in response to oral supplementation of type 2 Leprdb obese diabetic mice with niacin-bound chromium. Physiol. Genomics.

[78] Kocaefe YC, Israeli D, Ozguc M, Danos O, Garcia L (2005). Myogenic program induction in mature fat tissue (with MyoD expression). Exp. Cell Res.

[79] Comi GP, Fortunato F, Lucchiari S, Bordoni A, Prelle A, Jann S, Keller A, Ciscato P, Galbiati S, Chiveri L, Torrente Y, Scarlato G, Bresolin N (2001). Beta-enolase deficiency, a new metabolic myopathy of distal glycolysis. Ann. Neurol.

[80] Baranova A, Collantes R, Gowder SJ, Elariny H, Schlauch K, Younoszai A, King S, Randhawa M, Pusulury S, Alsheddi T, Ong JP, Martin LM, Chandhoke V, Younossi ZM (2005). Obesity-related differential gene expression in the visceral adipose tissue. Obes. Surg.

[81] Moore JW, Maher MA, Banz WJ, Zemel MB (1998). Chromium picolinate modulates rat vascular smooth muscle cell intracellular calcium metabolism. J. Nutr.

[82] McCarty MF (2006). PKC-mediated modulation of L-type calcium channels may contribute to fat-induced insulin resistance. Med. Hypotheses.

[83] Eyre H, Akkari PA, Wilton SD, Callen DC, Baker E, Laing NG (1995). Assignment of the human skeletal muscle alpha-tropomyosin gene (TPM1) to band 15q22 by fluorescence in situ hybridization. Cytogenet. Cell Genet.

[84] Ruiz-Opazo N, Weinberger J, Nadal-Ginard B (1985). Comparison of alpha-tropomyosin sequences from smooth and striated muscle. Nature.

[85] Gordon AM, Homsher E, Regnier M (2000). Regulation of contraction in striated muscle. Physiol. Rev.

[86] Stocker A (2004). Molecular mechanisms of vitamin E transport. Ann. N. Y. Acad. Sci.

[87] Bjornson LK, Gniewkowski C, Kayden HJ (1975). Comparison of exchange of alpha-tocopherol and free cholesterol between rat plasma lipoproteins and erythrocytes. J. Lipid Res.

[88] Traber MG, Burton GW, Hamilton RL (2004). Vitamin E trafficking. Ann. N. Y. Acad. Sci.

[89] Cinti S (2002). Adipocyte differentiation and transdifferentiation: plasticity of the adipose organ. J. Endocrinol. Invest.

[90] Zhou Z, Yon Toh S, Chen Z, Guo K, Ng CP, Ponniah S, Lin SC, Hong W, Li P (2003). Cidea-deficient mice have lean phenotype and are resistant to obesity. Nat. Genet.

[91] Roy S, Rink C, Khanna S, Phillips C, Bagchi D, Bagchi M, Sen CK (2004). Body weight and abdominal fat gene expression profile in response to a novel hydroxycitric acid-based dietary supplement. Gene Expr.

[92] Li C, Wong WH (2001). Model-based analysis of oligonucleotide arrays: expression index computation and outlier detection. Proc. Natl. Acad. Sci. USA.

[93] Ohia SE, Awe SO, LeDay AM, Opere CA, Bagchi D (2001). Effect of hydroxycitric acid on serotonin release from isolated rat brain cortex. Res. Commun. Mol. Pathol. Pharmacol.

[94] Langin D, Dicker A, Tavernier G, Hoffstedt J, Mairal A, Ryden M, Arner E, Sicard A, Jenkins CM, Viguerie N, van Harmelen V, Gross RW, Holm C, Arner P (2005). Adipocyte lipases and defect of lipolysis in human obesity. Diabetes.

[95] Roy S, Shah H, Rink C, Khanna S, Bagchi D, Bagchi M, Sen CK (2007). Transcriptome of primary adipocytes from obese women in response to a novel hydroxycitric acid-based dietary supplement. DNA Cell Biol.

[96] Garcia A, Sekowski A, Subramanian V, Brasaemle DL (2003). The central domain is required to target and anchor perilipin A to lipid droplets. J. Biol. Chem.

[97] Lin J, Handschin C, Spiegelman BM (2005). Metabolic control through the PGC-1 family of transcription coactivators. Cell Metab.

[98] Kim BW, Choo HJ, Lee JW, Kim JH, Ko YG (2004). Extracellular ATP is generated by ATP synthase complex in adipocyte lipid rafts. Exp. Mol. Med.

[99] Gloerich J, Ruiter JP, van den Brink DM, Ofman R, Ferdinandusse S, Wanders RJ (2006). Peroxisomal trans-2-enoyl-CoA reductase is involved in phytol degradation. FEBS Lett.

[100] Schluter A, Yubero P, Iglesias R, Giralt M, Villarroya F (2002). The chlorophyll-derived metabolite phytanic acid induces white adipocyte differentiation. Int. J. Obes. Relat. Metab. Disord.

[101] Jaye M, Lynch KJ, Krawiec J, Marchadier D, Maugeais C, Doan K, South V, Amin D, Perrone M, Rader DJ (1999). A novel endothelial-derived lipase that modulates HDL metabolism. Nat. Genet.

[102] Ahmed W, Orasanu G, Nehra V, Asatryan L, Rader DJ, Ziouzenkova O, Plutzky J (2006). High-density lipoprotein hydrolysis by endothelial lipase activates PPARalpha: a candidate mechanism for high-density lipoprotein-mediated repression of leukocyte adhesion. Circ. Res.

[103] Westerberg R, Tvrdik P, Unden AB, Mansson JE, Norlen L, Jakobsson A, Holleran WH, Elias PM, Asadi A, Flodby P, Toftgard R, Capecchi MR, Jacobsson A (2004). Role for ELOVL3 and fatty acid chain length in development of hair and skin function. J. Biol. Chem.

[104] Westerberg R, Mansson JE, Golozoubova V, Shabalina IG, Backlund EC, Tvrdik P, Retterstol K, Capecchi MR, Jacobsson A (2006). ELOVL3 is an important component for early onset of lipid recruitment in brown adipose tissue. J. Biol. Chem.

[105] Przybyla-Zawislak BD, Srivastava PK, Vazquez-Matias J, Mohrenweiser HW, Maxwell JE, Hammock BD, Bradbury JA, Enayetallah AE, Zeldin DC, Grant DF (2003). Polymorphisms in human soluble epoxide hydrolase. Mol. Pharmacol.

[106] Lee CR, North KE, Bray MS, Fornage M, Seubert JM, Newman JW, Hammock BD, Couper DJ, Heiss G, Zeldin DC (2006). Genetic variation in soluble epoxide hydrolase (EPHX2) and risk of coronary heart disease: The Atherosclerosis Risk in Communities (ARIC) study. Hum. Mol. Genet.

[107] Wei Q, Doris PA, Pollizotto MV, Boerwinkle E, Jacobs DR Jr, Siscovick DS, Fornage M (2007). Sequence variation in the soluble epoxide hydrolase gene and subclinical coronary atherosclerosis: interaction with cigarette smoking. Atherosclerosis.

[108] Dannewitz B, Edrich C, Tomakidi P, Kohl A, Gabbert O, Eickholz P, Steinberg T (2006). Elevated gene expression of MMP-1, MMP-10, and TIMP-1 reveal changes of molecules involved in turn-over of extracellular matrix in cyclosporine-induced gingival overgrowth. Cell Tissue Res.

[109] Figueroa J, Vijayagopal P, Debata C, Prasad A, Prasad C (1999). Azaftig, a urinary proteoglycan from a cachectic cancer patient, causes profound weight loss in mice. Life Sci.

[110] Obunike JC, Sivaram P, Paka L, Low MG, Goldberg IJ (1996). Lipoprotein lipase degradation by adipocytes: receptor-associated protein (RAP)-sensitive and proteoglycan-mediated pathways. J. Lipid Res.

[111] Maquoi E, Demeulemeester D, Voros G, Collen D, Lijnen HR (2003). Enhanced nutritionally induced adipose tissue development in mice with stromelysin-1 gene inactivation. Thromb. Haemost.

[112] Sasaki K, Takagi M, Konttinen YT, Sasaki A, Tamaki Y, Ogino T, Santavirta S, Salo J (2007). Upregulation of matrix metalloproteinase (MMP)-1 and its activator MMP-3 of human osteoblast by uniaxial cyclic stimulation. J. Biomed. Mater. Res. B Appl. Biomater.

[113] Lijnen HR, Van HB, Frederix L, Rio MC, Collen D (2002). Adipocyte hypertrophy in stromelysin-3 deficient mice with nutritionally induced obesity. Thromb. Haemost.

[114] Chang S, Young BD, Li S, Qi X, Richardson JA, Olson EN (2006). Histone deacetylase 7 maintains vascular integrity by repressing matrix metalloproteinase 10. Cell.

[115] Cao Y (2007). Angiogenesis modulates adipogenesis and obesity. J. Clin. Invest.

[116] Van Guilder GP, Hoetzer GL, Smith DT, Irmiger HM, Greiner JJ, Stauffer BL, DeSouza CA (2005). Endothelial t-PA release is impaired in overweight and obese adults but can be improved with regular aerobic exercise. Am. J. Physiol. Endocrinol. Metab.

[117] Friedman JM, Halaas JL (1998). Leptin and the regulation of body weight in mammals. Nature.

[118] Friedman JM (1998). Leptin, leptin receptors, and the control of body weight. Nutr. Rev.

[119] Cohen P, Zhao C, Cai X, Montez JM, Rohani SC, Feinstein P, Mombaerts P, Friedman JM (2001). Selective deletion of leptin receptor in neurons leads to obesity. J. Clin. Invest.

[120] Wittert GA, Turnbull H, Hope P, Morley JE, Horowitz M (2004). Leptin prevents obesity induced by a high-fat diet after diet-induced weight loss in the marsupial S. crassicaudata. Am. J. Physiol. Regul. Integr. Comp. Physiol.

[121] Nijhuis J, Van Dielen FM, Buurman WA, Greve JW (2004). Leptin in morbidly obese patients: no role for treatment of morbid obesity but important in the postoperative immune response. Obes. Surg.

[122] Liu-Stratton Y, Roy S, Sen CK (2004). DNA microarray technology in nutraceutical and food safety. Toxicol. Lett.

